# Constructing a Clinical Patient Similarity Network of Gastric Cancer

**DOI:** 10.3390/bioengineering11080808

**Published:** 2024-08-09

**Authors:** Rukui Zhang, Zhaorui Liu, Chaoyu Zhu, Hui Cai, Kai Yin, Fan Zhong, Lei Liu

**Affiliations:** 1 Institute of Biomedical Sciences, Fudan University, 131 Dongan Road, Shanghai 200032, China; 2 Department of Gastrointestinal Surgery, Changhai Hospital, Naval Military Medical University, 168 Changhai Road, Shanghai 200433, China; 3 Intelligent Medicine Institute, Fudan University, 131 Dongan Road, Shanghai 200032, China

**Keywords:** cPSN, data integration, gastric cancer, one-hot encoding, patient similarity calculation

## Abstract

Objectives: Clinical molecular genetic testing and molecular imaging dramatically increase the quantity of clinical data. Combined with the extensive application of electronic health records, a medical data ecosystem is forming, which calls for big-data-based medicine models. We tried to use big data analytics to search for similar patients in a cancer cohort, showing how to apply artificial intelligence (AI) algorithms to clinical data processing to obtain clinically significant results, with the ultimate goal of improving healthcare management. Methods: In order to overcome the weaknesses of most data processing algorithms that rely on expert labeling and annotation, we uniformly adopted one-hot encoding for all types of clinical data, calculating the Euclidean distance to measure patient similarity and subgrouping via an unsupervised learning model. Overall survival (OS) was investigated to assess the clinical validity and clinical relevance of the model. Results: We took gastric cancers (GCs) as an example to build a high-dimensional clinical patient similarity network (cPSN). When performing the survival analysis, we found that Cluster_2 had the longest survival rates, while Cluster_5 had the worst prognosis among all the subgroups. As patients in the same subgroup share some clinical characteristics, the clinical feature analysis found that Cluster_2 harbored more lower distal GCs than upper proximal GCs, shedding light on the debates. Conclusion: Overall, we constructed a cancer-specific cPSN with excellent interpretability and clinical significance, which would recapitulate patient similarity in the real-world. The constructed cPSN model is scalable, generalizable, and performs well for various data types.

## 1. Introduction

There are approximately 19.3 million new cancer cases diagnosed worldwide every year. Gastric cancer is the cancer with the fifth highest incidence and the fourth highest mortality, and about two-thirds of all cases are found in East Asia and Southeast Asia [1,2]. Achieving precision medicine for gastric cancer will hinge on the viability of big data analytics and AI models rooted in gastric cancer clinical data. While AI is widely used in biomedical science [3,4], medical data analysis models based on other diseases or algorithms adaptive for other conditions are ineffective in the field of tumors, which hinders the practical benefits of tumor clinical analytics. This is due to the fact that the granularity of clinical records is different in various diseases [5]. Tumor histopathological data as well as molecular and genetic data represent pivotal features with high information density and clinical value [6,7]. However, these two types of data are generally not involved in other diseases. Histopathological information mainly includes clinical descriptions, including imaging results, tumor site, tumor stage, differentiation, cellular composition, pathological type, and final diagnosis. Molecular data comprise marker expressions, genetic mutations, genomic features, and molecular classifications. Through pathological examination and molecular detection, in combination with other clinical data, tumor and tumor microenvironment characteristics can be comprehensively described and an exact diagnosis can be made, all of which underpin clinical decision-making. Moreover, precision medicine claims to be able to provide personalized therapy for every patient, fueled by clinical genetic testing because of the advances in cancer molecular genetics and genomics [8].

Even now, cancer continues to pose a great threat to human health. Inter-patient heterogeneity represents a great obstacle to cancer therapy. Conceivably, as cases of cancer are an enormous group, there are always some patients who are similar, and such historically similar patients may shed light on treatments for future patients. However, determining how to define and evaluate patient similarity remains controversial [9,10]. Patient similarity calculation, which assesses the similarity between patients by mathematically calculating data on the multi-modal heterogeneity metrics of patients, seems to be a solution. In general, the first step in patient similarity calculation is determining a multi-modal data processing and integration strategy. The second step is to define a similarity metric to calculate the distance or similarity score among patients in a systematic and consistent manner. The third step is to establish a patient similarity network (PSN) and carry out cluster analysis and clinical feature analysis in the PSN system. Finally, for patients to be evaluated, they would be embedded in the PSN and the group of patients most similar to the patient of interest would be defined based on the patient’s similarity score [11].

There have been some explorations into patient similarity calculations in human diseases [9,12]. They have generally used patient demographic information, diagnosis, treatment, prescription drugs, laboratory test data, and physiological monitoring data extracted from electronic medical records (EMRs). At present, some patient similarity calculations only use numerical variables as parameters to calculate Euclidean distance. This strategy presumes all variables are continuous, which is not perfectly suitable for categorical variables [13,14,15]. Some use the International Classification of Diseases (ICD) hierarchical coding to calculate the distance between the parent node and each child node for disease diagnoses and then evaluate the similarity [16,17], while some orchestrate medical record information into a medical knowledge graph and convert the medical entity relations into vector space, which can be used to calculate the Euclidean distance, Mahalanobis distance, or cosine similarity [18,19]. The method of encoding/embedding conversion has obvious defects as the information needs to be converted into other systems such as ICD coding and knowledge graphs, which are indirect calculations and bring various additional influencing factors, eventually affecting the accuracy of the results. The major drawback of patient similarity research is that it has struggled to incorporate diverse clinical data types into a unified model.

AI and deep learning have demonstrated usefulness in patient similarity analysis [20,21]. For example, disease characteristics are often mathematically represented as vectors or matrices, and neural networks are subsequently employed to learn similarities and cluster patients. However, models derived from neural networks are usually highly specialized [22]. Most patient similarity models based on supervised or semi-supervised algorithms are dependent on pre-labeled training data and require the extraction of parameters and corresponding exact weights. Although such a model performs well on an experimental dataset, the generalizability is weak. Even for the same disease, transferring an algorithmic model is difficult when the data metrics are different. This is a drawback of the supervised method [23]. Additionally, when defining and measuring patient similarity, data labeling is laborious and susceptible to subjective factors. In the era of biomedical big data [24], knowledge and decisions are obtained based on population data but not on the clinician’s experience, so the labeling is disgusted. These two drawbacks have led to the diminished clinical application value of deep learning represented by supervised neural networks in patient similarity assessment. Maybe self-organizing map is a promising neural network. 

Regarding research on patient similarity among gastric cancer patients, the topic is relatively understudied. One study developed a GC subtype classification model that integrates multi-omics fusion data and patient similarity networks via a residual graph convolutional network. However, their method was limited to handling numerical variables [25].

Our review suggests that identifying similar cases from a large pool of historical cancer patients, a process known as patient similarity analysis, holds great promise in clinical big data analysis. Nevertheless, the methods for performing patient similarity analysis are still in their infancy. The challenges are twofold: clinical data typically consist of both numerical and categorical variables, accompanied by a significant number of missing values, which demands an efficient data processing approach. Furthermore, the similarity of tumor patients remains a rough estimate, and high-quality labeled data are scarce. Fortunately, unsupervised learning techniques such as *K*-means and hierarchical clustering are well-suited to handle unlabeled data. To address these challenges, we developed a pipeline that leverages one-hot encoding and *K*-means clustering to construct a cancer-specific PSN. Then, the PSN was validated using survival endpoints or other indicators to ensure clinical validity. Our ultimate goal is to utilize the derived cPSN to facilitate patient stratification, uncover clinical characteristics, provide personalized treatment recommendations, and inform healthcare management.

## 2. Methods

### 2.1. Data Collection and Preprocessing

Multiple types of clinical data from one thousand patients with surgical GCs were collected from the department of gastrointestinal surgery, Shanghai Changhai Hospital. Clinical information was extracted from EMRs and medical examination reports, and the data were then preprocessed to ensure consistency in formatting. Clinical descriptions were summarized into keywords, such as classifying surgical procedures into laparotomy or laparoscopy. In terms of histopathological data, our dataset contained mesenteric vein/portal vein involvement, qualitative description of surgical margin status, tumor stage, tumor differentiation, etc. (Table 1). In terms of molecular genetic data, the dataset contained gene mutations derived from clinical genetic testing, gene expression, and immunohistochemical data. Emphatically, each gene mutation and each tumor marker/gene expression level can be considered as an independent variable. Missing data are marked with NA.

### 2.2. Encoding

In the encoding process, categorical variables were directly coded, numerical variables and clinical qualitative descriptions were converted into categorical variables, and each categorical state of each variable was recorded as a one-hot feature. Suppose that there are *M* observation indices (variables) in a set of samples, denoted as $X_{1},\;X_{2},\ldots ,\;X_{M}$, and each observation index $X_{i}$ has $N_{i}$ different classification states, denoted as $N_{1},\;N_{2},...,N_{M}$; altogether, we would obtain $\sum_{i=1}^{M}N_{i}$ one-hot features. Continuous values were transformed into discrete values by equivalent partitioning. Preferably, for numerical variables, the values in a set of samples were divided into 4 parts according to the quartile method so that 4 categorical variables were formed. For clinical qualitative descriptions, *N* states were formed into *N* categorical variables.

A missing value was regarded as an independent one-hot coding type in the observation index of clinical data, and there was no need to fill in null values.

The one-hot encoding method was engaged to integrate multi-modal medical data. Subsequently, the heterogeneous data of patients were transformed into a feature embedding matrix.

Through distance calculation (Euclidean distance in this study), the feature embedding matrix was organized into a PSN. Preferably, the *t*-distributed stochastic neighbor embedding (*t*-SNE) method can be used to visualize the high-dimensional network in a two-dimensional or three-dimensional display. 

### 2.3. Subgrouping

*K*-means clustering, an unsupervised learning algorithm, was conducted for the patient similarity analysis to divide all patients into *K* clusters. *K* is a hyperparameter that is set between 2 and 10. The elbow method or gap statistic method was used to evaluate the effect of clustering for each selected *K*. Data encoding and clustering analyses were conducted using scikit-learn packages in Python 3.10.

### 2.4. Survival Analysis

The Kaplan–Meier method was used for clinical endpoint correlation analysis. The log-rank test was used to assess the statistical differences in OS between different groups of patients following clustering. PSNs with or without clinical implications were obtained based on the statistical significance of *p*-values. If the *p*-value is less than 0.05, we would consider the constructed PSN to be correlated with a clinically meaningful endpoint, namely a cPSN. Survival analysis was conducted in R 4.0.3 using the survival and survminer packages.

### 2.5. Statistical Analysis

Statistical analyses were conducted using the chi-square test (*χ*^2^) in SPSS Statistics 20. Briefly, we created a contingency table to display the frequency of each classification (clustering group, patient age, cancer differentiation, or tumor stage). We examined the distribution of multiple categorical variables (dMMR, EGFR-IHC, ERBB2-IHC, p53-IHC) simultaneously and used a statistical test to determine if there is a significant association between the variables and subgroup, patient age, cancer differentiation, and tumor stage. A *p*-value of less than 0.01 indicates statistical significance.

## 3. Results

We collected multiple types of clinical data from 1000 patients with surgical GCs. In this study, the heterogeneous medical data we dealt with included demographic data, histopathological data, molecular and genetic data, laboratory tests, and the surgical paradigm narrative. The types of data contained numerical variables, binary variables, categorical variables, and clinical qualitative descriptions (Table 1).

Categorical data representation has advantages in capturing data from clinical records [26]. Numerical data is continuous values that are accurate, but this information does not necessarily have to be presented this way. Given that continuous values within a certain range could be considered to have similar clinical significance, and to improve the generalizability of the model, we transformed the continuous values into discrete values using equivalent partitioning. In this case, categorical variables were directly coded in the encoding process, and numerical variables and clinical qualitative descriptions were first converted into categorical variables. In order to integrate multi-modal medical data, we encoded the feature parameters of each patient using the one-hot encoding method. A total of 143 one-hot encoding values were identified from 37 variables, as a result of each categorical state of each variable being recorded as a one-hot feature. Subsequently, the heterogeneous data of patients were transformed into a feature embedding matrix.

**Figure 1:**
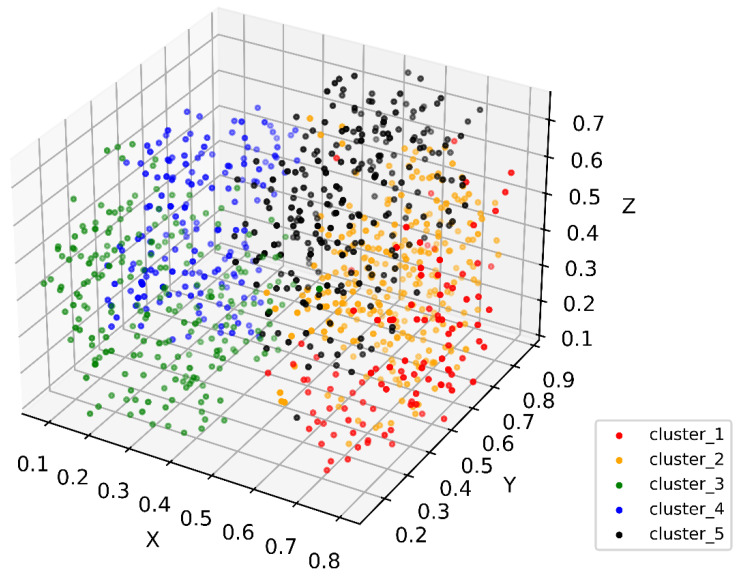
Three-dimensional *t*-SNE showing patient distribution in the constructed PSN. Colors show different subgroups identified in patient similarity analysis. Values in axes represent relative distance in the dimension.

**Figure 2:**
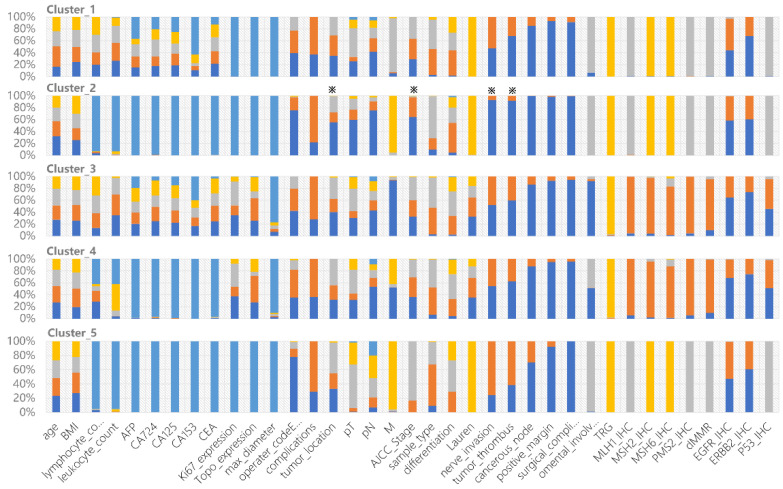
Clinical characteristics of each cluster derived from the constructed cPSN. The colors show the frequency of each categorical state of the variable. All 37 variables are shown in each cluster. ※ indicates specific features of Cluster_2 compared to other clusters.

Through feature coding, patient embedding, and distance calculation, all patient data were orchestrated to form a PSN, which is an *M*-dimensional network, where *M* is the sum of observation parameters. The PSN reflects the similarity distance between patients (Figure 1). Each point in the high-dimensional PSN represents a patient. We then conducted cluster analysis. The 1000 surgical GC patients were divided into 2 to 11 clusters via the *K*-means algorithm. Using the elbow method, five clusters were found to provide the best clustering performance. Each cluster represents a similar group sharing some clinical characteristics (Figure 2), which is the immanent foundation for treatment recommendations for a given patient who is clustered into a specific group.

**Figure 3:**
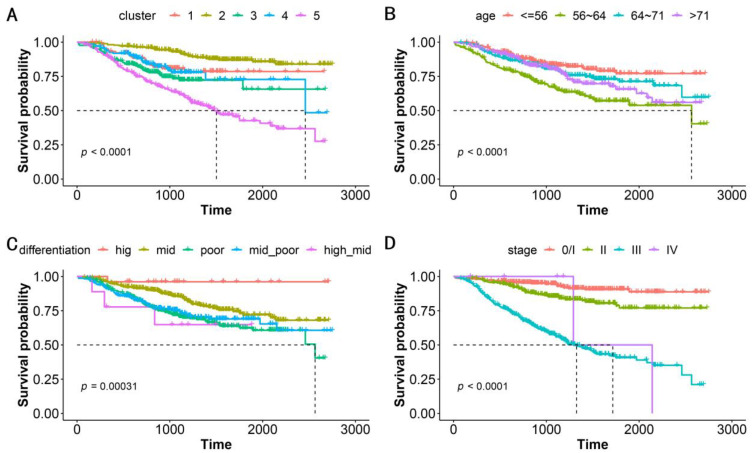
Kaplan–Meier survival analysis for OS by (**A**) subgroups, (**B**) patient age, (**C**) cancer differentiation, and (**D**) tumor stage. The five subgroups represent the patients classified into 5 clusters based on patient similarity calculation. Patient age is classified into quartiles. *p*-value shows statistical significance based on log-rank analysis.

We performed a correlation analysis of a clinically meaningful endpoint to evaluate the clinical validity of the clustering. OS, which serves as the gold standard of oncological clinical endpoints [27], was investigated to assess the validity and clinical relevance of the constructed PSN. When the patients in our cohort were divided into five clusters, the OS differences between clusters were statistically significant (log-rank test, *p* < 0.0001, Figure 3A). In addition to distinguishing this clinical endpoint, our clustering could also suggest specific gene mutations and genomic features in various subgroups (Table 2). Our strategy achieved an excellent performance that was superior to that using traditional classifications such as patient age, cancer differentiation, and tumor stage (Figure 3B–D, Table 2). Notably, ERBB2-IHC is related to differentiation, with the proportion of ERBB2-IHC positivity in patients with high, moderate-high, moderate, moderate-low, and low differentiation being 0.462, 0.556, 0.450, 0.322, and 0.219, respectively. This is the sole demonstration of an association between conventional classification approaches and genomic molecular features (Table 2).

Cluster_2 has the longest survival rates. Most patients in Cluster_2 are negative for nerve invasion, negative tumor thrombus, negative cancerous node, and regional nodal involvement. The patients are mainly in pathological stage I and stage II, with some scattered across other stages. All of these clinical indicators support better prognosis. Interestingly, we found that lower distal GCs are more common than upper proximal GCs, shedding light on the debates [28,29] (Figure 2). Cluster_5 has the worst prognosis among all subgroups. The patients are mainly in pathological stage III, with the majority having upper tumor locations. Cluster_1 contains 71.6% of patients of Mx, meaning that distant metastasis cannot be determined. TP53 mutations are predominantly found in Cluster_3 and Cluster_4, in accordance with their dMMR characteristics. The proportion of EGFR and ERBB2 expression is significantly lower in Cluster_3 and Cluster_4 (Figure 2).

## 4. Discussion

The present research provides a similarity calculation method for tumor patients based on one-hot encoding and unsupervised clustering. According to their clinical features, a cohort of tumor patients was embedded in a high-dimensional space and then clustered into several groups based on commonalities. We then assessed whether these different groups of patients were clinically distinct. While death is the primary event of interest in cancer patients, based on the OS of cancer patients, a correlation analysis of this clinical endpoint was carried out on the clustered patients. The log-rank test assessed statistical significance to examine whether the distribution of OS was distinguishable, which ensures the clinical significance and practical value of the established PSN. For example, cancer stage is conventionally used to stratify patients [30]. However, patients with different stages were often clustered into the same subset in our model. Furthermore, patients in the same subset tend to have similar survival prognoses, as well as potentially similar clinical characteristics and responses to treatment.

Clinical data resources include electronic medical records, imaging examinations, laboratory tests, and genetic and cellular analyses. Determining how to integrate highly heterogeneous patient data is vital in patient similarity analysis. We adopted the “early integration strategy”, which constructs a unified model for all types of data, in contrast to the “late integration strategy”, which calculates distances for each data type and requires searching for a corresponding appropriate model [31]. Note that the “early integration strategy” ignores the correlation between parameters. The Mahalanobis distance calculation, which weights multivariate parameters using a covariance matrix, may compensate for this shortcoming.

Data encoding was performed following the integration. We used the one-hot encoding strategy as it is concise and robust in clinical data management. It can efficiently code any clinical data, and the data processing ability is superior. As the parameters with clinical meaning and data accessibility are limited, dimensionality does not need to be considered. Although undesirable, missing data frequently occur in real-world healthcare scenarios when the values of variables are not measured or are unavailable for a patient. The usual practice is to fill in missing values with estimated values, which may underrepresent the real state, thus rendering them unsuitable for further analysis. The present study regarded missing values as independent one-hot coding types without filling in null values, which may reduce value bias and avoid the classification error caused by filling methods. 

Besides multi-modal medical data integration and encoding, data labeling in the field of tumor patient similarity lacks standards. Doctors’ annotations often rely on limited information and are usually based on heuristic judgements. These labeling processes are subjective or otherwise uncertain. However, AI algorithms and machine learning usually require a large amount of labeled data. This creates a great gap between the available manual labeling and the accurate labeling required for training algorithms. Unsupervised clustering, independent of any labeling data, efficiently classifies patients into subgroups. Thus, machine learning can then be used to uncover clinical characteristics or data features underlying the subgroups. Essentially, the constructed cPSN should authentically restore the similarity of patients in the real world, linking to prognostic assessment, personal treatment, and health management.

While a consensus on which machine learning algorithm performs better with specific data types in the context of precision medicine is still lacking [32], the present study performed *K*-means unsupervised clustering and evaluated *K* using a statistical algorithm to obtain the optimal *K*, and the whole process was unsupervised without human intervention. The present study uniformly adopted one-hot encoding for multi-modal, highly heterogeneous clinical data, which is flexibly compatible with clinical data evolvement and changes in observation status caused by different medical institutions, doctors, and medical development stages. The data processing method provides an extensibility mechanism for adding more parameters. In the future, when synthetic data are expected to replace real data in medical big data analytics [33], machine learning algorithms can be used to accelerate clinical trials, which is a subsequent mission of patient similarity analysis.

Our cPSN holds great clinical value in the context of cancer care management. This big data analytics approach can elucidate subtle clinical conundrums, including the disparities in prognosis between distal and proximal gastric cancer patients and the prognostic differences associated with ERBB2 expression in gastric cancer, as exemplified by our research findings. To evaluate target a patient, the group of patients most similar to the target index patient is identified in the cPSN using the *K*-nearest neighbor algorithm based on distance calculation, and the range and fineness of similar patients are selected by adjusting the *K* value. Then, therapeutic insights can be acquired from similar patients to help prognostic evaluation. Thus, population-based clinical information obtained by searching similar patient cases can be used to propose treatment and management strategies, which would promote the development of big-data-based precision medicine.

Altogether, we developed an easy-to-perform, clinically interpretable, generalizable, and universal method to conduct cancer patient similarity analysis. Our cPSN could create paths from clinical data to insight, and from information to decision. With an emphasis on clinical utility and usability, clinical investigators can use the cPSN to find insights and conduct clinical research. Clinicians can use the cPSN to inform patient stratification, recommend treatments, deliver personalized patient care, and improve population health management.

This study has several limitations. Firstly, although one-hot encoding is a robust method that can dispose of missing values, too many null values in the dataset affect the accuracy of the results. It is better to apply as complete records as possible in future studies. Secondly, the data we used were baseline data that depicted the patients’ features before surgery, without considering treatment information. That may make sense because a causal connection exists between baseline data and treatment programs. However, if treatment data are available, we may be able to discover whether patient outcomes are more influenced by baseline characteristics or treatment regimen/treatment sequence through patient similarity analysis. Thirdly, our model ignored the correlations among the parameters of selected features, leading to potential redundancy. We recognize that parameter redundancy and non-weight matrices are limitations of the cPSN. These require solutions, especially under the framework of unsupervised learning.

## 5. Conclusions

By integrating heterogeneous clinical data (e.g., histopathological data, molecular and genetic data, laboratory data, diagnosis, and treatment), we constructed a cPSN for gastric carcinoma patients that can recapitulate patient similarity in the real world. The constructed model is scalable, generalizable, and performs well for various data types. Moreover, our cPSN is associated with clinical implications, which could give researchers insights into clinical issues. The constructed cPSN could be used to accurately “locate” patients of interest, classify them into a disease subtype, support medical decision-making with reference to past similar cases, and predict clinical outcomes. In the future, prospective clinical trials are warranted to validate the clinical efficacy of the model.

## Figures and Tables

**Table 1:** Data summary of baseline characteristics of 1000 GC patients.

Parameter Name	Count	Parameter Type	Parameter Name	Count	Parameter Type
Median age (range)	64 (24–93)	Continuous	Nerve_invasion		Binary
Median BMI (range)	23.1 (14.2–54.1)	Continuous	Yes	433	
Median lymphocyte_count (range)	1.41 (0.12–3.81)	Continuous	No	567	
Median leukocyte_count (range)	6.4 (2.66–27.58)	Continuous	Tumor_thrombus		Binary
Median AFP (range)	2.46 (0.74–136.41)	Continuous	Yes	353	
Median CA724 (range)	2.2 (0.37–300)	Continuous	No	647	
Median CA125 (range)	10.7 (2.7–391.4)	Continuous	Cancerous_node		Binary
Median CA153 (range)	7 (2.7–20.2)	Continuous	Yes	138	
Median CEA	2.3 (0.5–1500)	Continuous	No	862	
Median Ki67_expression	0.6 (0.01–0.9)	Continuous	Positive_margin		Binary
Median Topo_expression	0.4 (0.01–0.9)	Continuous	Yes	138	
Median max_diameter	3 (0.8–18)	Continuous	No	862	
operater_codeEMR		Categorical	Surgical_complications		Binary
Laparotomy	585		Yes	31	
Laparoscope	283		No	969	
Laparoscopic_exploratory_surgery	120		Omental_involvement		Binary
NA	12		Yes	7	
Complications		Binary	No	295	
Yes	288		NA	698	
No	712		TRG		Categorical
Tumor_location		Categorical	1 grade	1	
Lower	408		2 grade	4	
Middle	222		3 grade	5	
Upper	360		NA	990	
Residual	10		MLH1_IHC		Categorical
pT		Categorical	(-)	20	
Tis/T1	309		(+)	363	
T2	112		NA	617	
T3	394		MSH2_IHC		Categorical
T4a	172		(-)	13	
T4b	13		(+)	357	
pN		Categorical	Little (+)	12	
N0	447		NA	618	
N1	162		MSH6_IHC		Categorical
N2	157		(-)	6	
N3a	149		(+)	320	
N3b	85		Little (+)	48	
M		Categorical	NA	626	
M0	299		PMS2_IHC		Categorical
M1	6		(-)	19	
Mx	134		(+)	363	
NA	561		NA	618	
AJCC_Stage		Categorical	dMMR		Binary
Stage 0/stage I	338		Yes	38	
Stage II	278		No	332	
Stage III	378		NA	630	
Stage IV	6		EGFR-IHC		Categorical
Sample_type		Categorical	(-)	586	
Proximal	68		(+)	406	
Total	409		(±)	8	
Distal	508		ERBB2-IHC		Categorical
Residual	15		(-)	663	
Differentiation		Categorical	(+)	337	
High	26		p53-IHC		Categorical
Middle	367		(-)	183	
Poorly	365		(+)	185	
Middle_poorly	233		NA	632	
High_middle	9				
Lauren		Categorical			
Intestinal type	128				
Diffuse type	124				
Mixed	76				
NA	672				

**Table 2:** Chi-square tests between the categorical variables and subgroups, patient age, cancer differentiation, and tumor stage.

Classification	dMMR	EGFR-IHC	ERBB2-IHC	p53-IHC
Clustering	0.000 *	0.000 *	0.001 *	0.000 *
Age	0.193	0.723	0.010	0.575
Differentiation	0.036	0.339	0.000 *	0.043
Stage	0.106	0.794	0.162	0.396

* Statistically significant.

## Data Availability

The data used to support the findings of this study are available from the corresponding author upon reasonable request.
